# COVID-19 in Brazil: Are there any differences in Mental Health Literacy between young and aged men?

**DOI:** 10.1590/1518-8345.5651.3544

**Published:** 2022-07-15

**Authors:** Wanderson Carneiro Moreira, Anderson Reis de Sousa, Rachel da Silva Serejo Cardoso, Aline Macêdo de Queiroz, Marcia Aparecida Ferreira de Oliveira, Carlos Alberto da Cruz Sequeira

**Affiliations:** 1 Universidade de São Paulo, Escola de Enfermagem, São Paulo, SP, Brasil.; 2 Universidade Federal da Bahia, Escola de Enfermagem, Salvador, BA, Brasil.; 3 Universidade Federal Fluminense, Escola de Enfermagem Aurora de Afonso Costa, Niterói, RJ, Brasil.; 4 Universidade Estácio de Sá, Curso de Enfermagem, Rio de Janeiro, RJ, Brasil.; 5 Universidade Federal do Pará, Faculdade de Enfermagem, Belém, PA, Brasil.; 6 Universidade do Porto, Escola Superior de Enfermagem do Porto, Porto, Portugal.

**Keywords:** Mental Health Literacy, Health Literacy, Mental Health, Men’s Health, Coronavirus Infections, Adult Health, Elderly Health, Letramento em Saúde, Saúde Mental, Saúde do Homem, Infecções por Coronavírus, Saúde do Adulto, Saúde do Idoso, Alfabetización en Salud, Salud Mental, Salud del Hombre, Infecciones por Coronavirus, Salud del Adulto, Salud del Anciano

## Abstract

**Objective:**

to analyze the Mental Health Literacy of young and aged men living in Brazil in the COVID-19 pandemic context.

**Method:**

a qualitative study conducted with 87 men by means of an asynchronous online survey. The data were processed in the NVIVO12^®^ software, structured with the Collective Subject Discourse method and analyzed through Anthony Jorm’s theoretical concept of Mental Health Literacy.

**Results:**

six central ideas emerged from the analysis of a synthesis discourse on the components of the Mental Health Literacy, namely: *Ability to recognize specific disorders or different types of psychological distress; Knowledge and beliefs about risk factors and causes; Knowledge and beliefs about self-help interventions; Knowledge and beliefs about available professional help; Attitudes that facilitate recognition and the search for adequate help; and Knowledge on how to seek information on mental health*.

**Conclusion:**

there are differences in the mental health literacy of young and aged men living in Brazil during the COVID-19 pandemic. Aged men were more competent for mental health care management and protection than young men, in relation to the Mental Health Literacy levels.

## Resumo

**Objetivo:**

analisar o Letramento em Saúde Mental de homens jovens e idosos residentes no Brasil no contexto da pandemia da COVID-19.

**Método:**

estudo qualitativo, realizado com 87 homens, por meio de *survey online* assíncrona. Os dados foram processados no *software* NVIVO12^®^, estruturados com o método do Discurso do Sujeito Coletivo e analisados por meio do conceito teórico de Letramento em Saúde Mental de Anthony Jorm.

**Resultados:**

a partir da análise de um discurso síntese sobre os componentes do Letramento em Saúde Mental, emergiram seis ideias centrais: *capacidade de reconhecer distúrbios específicos ou diferentes tipos de sofrimento psíquico; conhecimento e crenças sobre fatores e causas de risco; conhecimento e crenças sobre intervenções de autoajuda; conhecimento e crenças sobre a ajuda profissional disponível; atitudes que facilitem o reconhecimento e a procura de ajuda adequada; e conhecimento de como buscar informações sobre saúde mental*.

**Conclusão:**

há diferenças no Letramento em Saúde Mental de homens jovens e idosos residentes no Brasil em vivência da pandemia da COVID-19. Homens idosos mostraram-se mais competentes para a gestão do cuidado e a proteção em saúde mental do que os homens jovens, em relação aos níveis de Letramento em Saúde Mental.

## Resumen

**Objetivo:**

analizar la Alfabetización en Salud Mental de hombres jóvenes y mayores residentes en Brasil en el contexto de la pandemia de COVID-19.

**Método:**

estudio cualitativo, realizado con 87 hombres, mediante *survey online* asincrónica. Los datos fueron procesados usando el *software* NVIVO12^®^, estructurados usando el método del Discurso del Sujeto Colectivo y analizados usando el concepto teórico de Alfabetización en Salud Mental de Anthony Jorm.

**Resultados:**

del análisis de un discurso síntesis sobre los componentes de la Alfabetización en Salud Mental, surgieron seis ideas centrales: *capacidad para reconocer trastornos específicos o diferentes tipos de sufrimiento psíquico; conocimiento y creencias sobre factores y causas de riesgo; conocimiento y creencias sobre las intervenciones de autoayuda; conocimiento y creencias sobre la ayuda profesional disponible; actitudes que facilitan el reconocimiento y la búsqueda de ayuda adecuada; y conocimiento sobre cómo buscar información sobre salud mental*.

**Conclusión:**

hay diferencias entre la Alfabetización en Salud Mental de los hombres jóvenes y la de los mayores que vivían en Brasil durante la pandemia de COVID-19. Los hombres mayores fueron más competentes para gestionar el cuidado y proteger su salud mental que los hombres jóvenes, según los niveles de Alfabetización en Salud Mental.

**Highlights:**

(1) This is the first study on male Mental Health Literacy and COVID-19.

(2) Aged men have higher MHL levels than young men.

(3) Young men can be more vulnerable to mental illness during the pandemic.

(4) Increasing the MHL levels assists in prevention, early intervention and self-help.

(5) Nursing can assume a leadership role in the promotion of MHL.

## Introduction

Due to the effects on mental health of the global population caused by the COVID-19 (Coronavirus Disease 2019) pandemic, a significant number of people became mentally ill. A number of studies indicate a high prevalence of mental disorders such as anxiety, depression, loneliness, sleep disorders, phobias, panic and post-traumatic stress in the population^1^ ^-^ ^3^. Regarding gender, data on men’s mental health in the COVID-19 pandemic context^4^ ^-^ ^5^ are still discrete.

In this sense, knowledge of the preventive measures related to mental health and recognition of the symptoms and treatment modalities for mental disorders is fundamental, in addition to enabling support to people suffering these conditions^6^. This perceived behavior is defined as Mental Health Literacy (MHL). Although little discussed in Brazil, MHL has been emerging in health promotion, mainly in view of the vulnerability related to mental health imposed in the pandemic context^7^.

The MHL concept emerged in Australia in the late 1990s^8^, where authors defined it as the knowledge and beliefs about mental disorders that assist in their recognition, treatment and prevention. Since then, researchers around the world have shown a growing interest in this phenomenon, leading to the evolution of the conceptual definition of MHL. Currently, MHL refers to the knowledge and skills required to promote mental health^9^, and it is divided into four components, namely: understanding how to achieve and maintain good mental health, understanding mental disorders and their treatments, reducing the stigma related to mental disorders, and increasing the effectiveness of the search for help^10^ ^-^ ^11^.

Low MHL levels exert an influence on the treatment-seeking behavior and self-management of the population’s psychosocial problems, reason why it is relevant that health professionals act assertively in the promotion of MHL^12^ ^-^ ^13^. With regard to gender, the stereotyped culture of the male model of strong active men who do not get sick still prevails in the Brazilian social construction^12^. “Hegemonic masculinity” can be an impediment to high MHL levels in this population segment, as men tend to complain less, deny pain and weakness, and hide physical and psycho-emotional frailty^13^. There is diverse evidence that men only seek help when pain becomes unbearable or incapacitating and when it affects work performance^12^.

As for the age-related MHL barriers, older adults gain prominence in the pandemic for presenting changes resulting from senescence and senility, but mainly due to the potentiation of *ageism* and stigmatizing attitudes to aged individuals by part of the population. In addition to that, necessary measures such as social distancing become important to prevent spread of the virus, but bring about complications to the routine habits and mental health of this group^14^.

Infodemics also have important repercussions for the mental health of the population, mainly that of older adults^15^. It is verified that, at a moment like this, full of uncertainties and excesses of information in all forms, including fake news, a rapid change in the world population’s behavior is necessary to reduce the impacts generated to mental health in the face of COVID-19^16^. Therefore, adequate MHL levels have never been as important as they are today.

From this perspective, by recognizing that this problem confers a significant attention focus to global public health, it is recommended to strengthen the strategies for mental health protection and promotion^17^ ^-^ ^18^, both during the course of the pandemic and in the post-pandemic period, with maximization of the MHL levels across the populations. Therefore, it is understood that developing MHL can prevent acute and chronic mental health problems and protect men from adopting toxic masculinity models.

The COVID-19 pandemic warned about the existing gaps in the MHL levels of the male population and exposed the need for an analysis to identify the state of this phenomenon in the countries, especially those where this topic has not yet been addressed, such as Brazil. Thus, the guiding question of the study was the following: How is the MHL of young and aged men configured in the COVID-19 pandemic context in Brazil? Given this question, this study aims at analyzing the MHL of young and aged men living in Brazil in the COVID-19 pandemic context.

## Method

### Study design

A qualitative study, structured in discursive analysis^19^ and following the Consolidated Criteria for Reporting Qualitative Research (COREQ) guidelines^20^.

### Scenario and participants

The study was carried out in a virtual environment, in all 26 Brazilian states and in the Federal District, Brazil, between June and September 2020, a period in which social restriction measures were established, recommended by governmental and health authorities to control spread of COVID-19.

A total of 87 men participated in the study (53 young and 34 aged). Cisgender and transgender men were included, over 18 years old and living in Brazil. Tourists were excluded. To define the age group, the World Health Organization (WHO) classification for young (from 25 to 44 years old) and aged (from 60 to 90 years old) subjects was used^21^.

Following the snowball sampling criteria^22^, two men from each region of the country were recruited for the study, through digital social networks (Facebook^®^, Instagram^®^ and WhatsApp^®^), one young and one aged *per* region, totaling 10 participants (we chose the most populous state in each of the five Brazilian regions). These were the first participants and were called “seeds”. Each participant received a link containing the Informed Consent Form in virtual format and the research form and was instructed to invite other men from their social network (without interference from the researchers) until a significant sample was obtained.

### Data collection and analysis

Data collection was carried out through an online survey, with application of a semi-structured instrument via the Google Forms^®^ digital platform*.* The form comprised closed questions on sociodemographic characterization, which contained variables such as: gender identity, sexual orientation, race/skin color, age, income, marital status, schooling, region and place of residence; as well as four open questions that dealt with knowledge and care strategies related to mental health, namely: 1. In your opinion, how does the pandemic affect male mental health? Tell me about it; 2. In relation to your mental health, describe what the pandemic has meant to you; did it cause you any problems? 3. How have you been feeling during the pandemic? Describe your feelings and emotions; 4. Tell us what you did to take care of you mental health and face problems related to it. The content of the instrument was reviewed and validated by three experts external to the research group.

Data collection was terminated when theoretical data saturation was reached, where, as new testimonies were included, two researchers independently carried out a continuous data analysis process through compilation and grouping of the topics identified^23^. This double analysis allowed identifying similar topics, with theoretical saturation achieved in 53 testimonies of the young individuals and 34 statements of the aged subjects, represented by the absence of new elements in the treated material, data co-occurrence, convergence and complementarity^24^. A third researcher reviewed the material and ensured theoretical saturation by analyzing the theoretical density and reliability of the results.

Data coding was supported by the NVIVO12^®^ software, with organization in charge of two researchers, which made it possible to process the material, create “nodes” - thematic codes, organize, identify and systematize categories and subcategories based on identified topics^25^.

The methodological analysis was structured by the Collective Subject Discourse (CSD) technique, which is configured in a form of social representation, in which common sense knowledge is present in the opinions and also in the stances adopted by a subject in everyday life^26^ ^-^ ^27^. This form of social representation can be understood as a synthesis of a stranger, approaching empirical reality. Therefore, in this study, line-by-line reading of the data was used, as well as location of discursive fragments and their relationships, convergences and complementarities, as a way to derive the Key Expressions, subsequently grouping the data, in the search for the Central Ideas and their anchorages. Finally, the synthesis-testimonies were structured, from the junction of the individual testimonies, written in the first person singular, in order to express the collective group under study, in consideration of men’s representation^19^ ^,^ ^26^ ^-^ ^27^. 

As this was the investigation of a single phenomenon with different population segments (young and aged men), the same methodological technique was used^19^, complying with scientific rigor, as a way of apprehending and explaining the specificities found in each age group. In this sense, a Synthesis-Discourse of structuring character was evidenced, comprised by six Central Ideas: 1. Ability to recognize specific disorders or different types of psychological distress; 2. Knowledge and beliefs about risk factors and causes; 3. Knowledge and beliefs about self-help interventions; 4. Knowledge and beliefs about available professional help; 5. Attitudes that facilitate recognition and the search for adequate help; and 6. Knowledge on how to seek information on mental health. 

The Central Ideas emerged from junction of the data, from the location of the collective discourse - data of greater expressiveness - density, deepening and representation of the investigated reality^26^, presented in both groups under study, expressed in the study in a separate way, namely: Young men’s CSD and aged men’s CSD.

The Synthesis-Discourse was subsidized by the anchorage^27^, derived from the explanation provided by the Central Ideas. Thus, the CSD obtained the representation that there are different MHL levels among men, based on their age group, manifested in the pandemic context (Figure 1).

**Figure 1:** Analytical structuring of apprehension of the Collective Subject Discourse of young and aged men living in Brazil about Mental Health Literacy in the COVID-19 pandemic context. São Paulo, SP, Brazil, 2021

CSD: “From management to protection: Explaining the distinctions in the Mental Health Literacy components for young and aged men.”		
**Methodological figures of the CSD**	**Group of study participants**	
Key Expressions:	G01-Young Men: [...] *mental health has been fluctuating during the pandemic* [...] *acceleration of thought and agitation* [...] *I was more concerned with the risk of contagion* [...] *I started to get into contact with close friends* [...] *I counted on the help of psychologists* [...] *practicing physical activity* [...] *I started to shield myself from having access to bad news* [...]*.*	G02-Aged Men: [...] *pressure and stress* [...] *it gradually increased the concerns and generated malaise* [...] *I tried to acquire more knowledge about the subject matter* [...] *I resorted to services offered on the Internet* [...] *search for contemplation, meditation, access to art, self-knowledge* [...] *getting updated through reports on television, on official websites of health agencies* [...]*.*
Central Ideas:	G01-Young Men: [...] *I started to have anxiety and panic crises* [...] *I needed to use medications* [...] *I imagined that the pandemic wouldn’t be so serious as to affect my mental health* [...] *I was trying to better understand the disease* [...] *to help me cope better with the increase in anxiety, fear and concern* [...] *generate well-being* [...] *witnessing so many social problems, which only worsen my mental state*[...]*.*	G02-Aged Men: [...] *I started to witness positive cases in co-workers* [...] *the emergence of tensions in relation to work, stress, it affected my mental health* [...] *I started to live with the uncertainty of the unexpected* [...] *I sought to understand the reality of what was happening* [...] *trying to solve problems that were affecting my mental health* [...] *a positive and effective strategy to keep my mental health balanced* [...] *reading was essential for me to perform actions that improved care for my mental health* [...]*.*
Anchorages:	G01-Young Men: They devote less attention to mental health management and protection.	G02-Aged Men: They are concerned with mental health management and protection and put into practice.
Synthesis-Testimonies:	1. Ability to recognize specific disorders or different types of psychological distress; 2. Knowledge and beliefs about risk factors and causes; 3. Knowledge and beliefs about self-help interventions; 4. Knowledge and beliefs about available professional help; 5. Attitudes that facilitate recognition and the search for adequate help; 6. Knowledge on how to seek information on mental health.	

After the methodological analysis, an analysis and/or theoretical interpretation of the material was performed, supported by the theoretical framework proposed by Anthony Jorm, which conceptualizes and structures the MHL components^10^ ^-^ ^11^ ^,^ ^28^.

### Legal and ethical aspects

Approval was obtained from the National Research Ethics Commission and the Research Ethics Committee of the Federal University of Bahia, under opinion number: 4,087,611, respecting all national and international criteria of ethics in research involving human beings. Anonymity of the participants was ensured, with identification of the testimonies by the initials “CSD” (Collective Subject Discourse) followed by the corresponding age group (Young Men or Aged Men).

## Results

The study included 87 men living in Brazil (53 young and 34 aged) between 25 and 33 years old (young) and from 63 to 72 years old (aged). The young men were mostly of cisgender gender identity and heterosexual sexual identity, characteristics similar to those of the aged men. In relation to race/skin color and marital status, most of the young men self-declared as brown-skinned and single, while the aged subjects mostly self-declared as white-skinned and married.

Both groups presented higher education level and work contracts in the public service. They reported having different incomes: between 3 and 4 minimum wages in the young men and above 5 minimum wages in the aged subjects. The men’s territory of residence was also different: the young men mostly lived in the Northeast region, with a higher concentration in the states of Bahia and Pernambuco, and the aged men were from the Brazilian Southeast region, with a higher concentration in the states of São Paulo and Rio de Janeiro. The young men lived with family members (parents and/or siblings), while the aged subjects lived with their spouses, and both live in urban areas.

## Synthesis-Discourse: From management to protection: Explaining the distinctions in the Mental Health Literacy components of young and aged men

The male experience in the everyday experience of the COVID-19 pandemic in Brazil explained the CSD of young and aged men that brought to light the MHL components, which are supported by six discursive categories of Central Ideas, described below. 

*Central Idea 1: Ability to recognize specific disorders or different types of psychological distress:*

*[...] I’ve noticed that my mental health has been fluctuating during the pandemic. After the virus reached Brazil, I started to have anxiety and panic crises, which rose mainly at the beginning of March. I had episodes of insomnia, which was caused by acceleration of thought and agitation and, because of that, I needed to use medications to sleep at night. I’ve experienced a constant sensation of discomfort and imminent danger.* (Young men’s CSD).

*[...] I had changes in my behavior and in my mental health status. My mental health became more affected when I began to witness positive cases in co-workers, increasing pressure and stress. Other problems have contributed to altering my mental health, such as the fear of being contaminated by the Coronavirus and having COVID-19 when I need to go to the street, my risk condition, as I have diseases and the uncertainty that generates impacts, making me more distressed and sad.* (Aged men’s CSD).

*Central Idea 2: Knowledge and beliefs about risk factors and causes*

*[...] before I imagined that the pandemic wouldn’t be so serious as to affect my mental health. As I’m young, I didn’t imagine taking greater risks, nor did I become shaken or mentally ill. I confess that I was more concerned about the risk of contagion and the problems that could make me financial unstable, but not about having problems with my mental health that could be the result of negative repercussions caused by anxiety, for example. Because of this, it took me a long time to believe in existence of the disease and to adhere to the quarantine. However, I started to feel afraid of the disease, of being infected by the virus, and of having my mental health compromised not only by COVID-19, but by the consequences that the disease could cause me.* (Young men’s CSD).

*[...] I thought that the pandemic couldn’t harm my mental health, but as the emergence of tensions in relation to work in the face of the high risk of contamination, overload and stress, the fear of having to move in public transportation, was increasing concerns and generating malaise, which affected my mental health. I started to feel uncomfortable in places with many people close to me, for fear of being contaminated and suffering from the disease, especially with the fact of living with the uncertainty of the unexpected. Stress, tension, confinement and social isolation have been the main causes of problems for my mental health. I didn’t imagine that I could feel anxious because of the pandemic, but I ended up realizing this situation.* (Aged men’s CSD).

*Central Idea 3: Knowledge and beliefs about self-help interventions*

*[...] I gradually sought to better understand the disease, what could cause arrival of the pandemic in Brazil and what it could generate in relation to impacts on my mental health. I started to get into contact with close friends to obtain and also exchange information that would help me to establish strategies to reduce the impacts on my health, such as, for example, strengthening the bonds of friendship, companionship, presence, mutual support, as I was unable to be with them, due to social isolation, which was causing me a lot of loneliness. The exchange of experience with friends and people in my coexistence cycle, such as work, helped me a lot to experience difficult situations and problems.* (Young men’s CSD).

*[...] with the emergence of the pandemic in Brazil, and more specifically in my city, I sought to understand the reality of what was happening and soon tried to obtain more knowledge about the subject matter, read about it in reliable sources, in order to think about how I should act in the face of everything that was being reported on television, on the Internet and through WhatsApp messages. When I started to feel the negative effects of the pandemic on my mental health, mainly due to the increase in tension and physical changes in my body, which lasted for several days, and the fear that the situation could get even worse, I started to devise some individual planning to adapt to the pandemic and protect my mental health, the health of my family members, my finances and in an attempt to seek to learn and overcome the transformations imposed by the pandemic on my life. However, this isn’t an easy task, because as a man I’m trained by society never to show weakness, nor to give up, which prevents me from exposing my feelings and my real mental health situation.* (Aged men’s CSD).

*Central Idea 4: Knowledge and beliefs about available professional help*

*[...] in order to face the problems with my mental health caused by the pandemic I had the help of psychologists, through online therapy. I watched some “lives” with holistic psychologists and therapists to help me deal better with the increase in anxiety, fear and concern about COVID-19, I became depressed and needed to seek the psychiatrist, and started using medications.* (Young men’s CSD).

*[...] I resorted to services offered on the Internet with the purpose of obtaining psychological support, sharing experiences, trying to solve problems that were affecting my mental health. I participated in virtual therapeutic groups with men that stimulated me to develop activities on a daily basis, such as leisure, entertainment, anxiety and stress control, relaxation practices, decreased consumption of information about COVID-19.* (Aged men’s CSD).

*Central Idea 5: Attitudes that facilitate recognition and the search for adequate help*

*[...] I’ve sought to carry out activities that do me good and generate well-being such as leaving the work routine, eating what gives me pleasure, practicing physical activity and strengthening relationships, as I know that all of this can improve my mental health.* (Young men’s CSD).

*[...] the search for contemplation, meditation, access to art, search for self-knowledge, reflection on the pandemic gives me a positive and effective strategy to keep my mental health balanced, as they reduce my anguish and anxiety levels. I stopped thinking about life, because I know that it’s important to reflect on attitudes, to know how to act with people and with life, it’s a real self-assessment, learning that is worth a lifetime. In addition to that, the search for God, because I know that He is in control of everything and makes me more confident, hopeful that everything will be over and improve.* (CSD for elderly men).

*Central Idea 6: Knowledge on how to seek information on mental health*

*[...] as I imagined that the pandemic could affect my mental health, disturbing my sleep, I went looking for psychological help. In addition to that, I started to shield myself from having access to bad news related to the fight against the pandemic in Brazil, given the government’s weaknesses. So I chose to consult other information sources, such as close friends, and thus reduce the stress, irritation and revolt of witnessing so many social problems, which only worsen my mental state.* (Young men’s CSD).

*[...] I started to read a lot about the pandemic and getting updated through reports on television, official websites of health agencies, scientific articles, and also reliable information passed on by close friends through WhatsApp. Reading was essential for me to perform actions that improved care for my mental health.* (Aged men’s CSD).

## Discussion

This study revealed the MHL components of young and aged men in the COVID-19 pandemic context in Brazil. Our results evidenced different MHL levels among the generations of men under study, from the exposure of knowledge and beliefs related to mental health problems, linked to the way in which men implement the self-help interventions, recognize the need for available professional help, build attitudes and seek diverse information for mental health care management in the COVID-19 pandemic context.

The ability to recognize specific disorders or different types of psychological distress pointed to the perception about the symptoms, causative agents, repercussions and impacts generated to mental health. Our results showed the self-knowledge of the individual and collective situation/condition of men’s mental health in the epidemic routine of a new communicable disease. This ability to recognize disorders and psychological distress was differentiated between the men’s age groups, with worsening due to the experience of anxiogenic processes, panic, stress, hypervigilance, psychomotor agitation and alteration of the sleep pattern being observed in the young men’s CSD, while the aged subjects recognized more comprehensive dimensions and not so centered on a single problem (the pandemic), but on the behavioral aspects, socio-affective interaction, work, vulnerabilities of the individual health condition due to risk factors - presence of chronic diseases and health-related fears resulting from illness by COVID-19.

Such representative findings contribute to explaining the fact that the young men’s experiences are anchored in the weak presence of the MHL levels manifested in the COVID-19 epidemic everyday life, when compared to those of the aged men, who, at the heart of the health crisis, proved to be more competent in dealing with the management of emotions and feelings. 

Properly recognizing mental health problems is an important factor that raises the MHL levels. If there are failures in this process, communication problems with health professionals may arise, considering that detection of a mental disorder is greater when people present their symptoms as a reflection of a psychological problem, not going unnoticed by general practitioners. Although clinical recognition may not be sufficient by itself to benefit the patients, it is a first step towards effective action^8^ ^-^ ^11^ ^,^ ^28^.

The presence of erroneous stereotypes regarding mental health, in relation to the adoption of early care and protection measures, was found in this study. The young men showed slight concern related to their mental health situation in the face of the pandemic, even though they recognized risk factors and causes for mental health and have harmful repercussions, which leads to certain disbelief in the phenomenon (pandemic) and in its severity and complexity for mental health. Such beliefs, probably intensified by the action of gender hegemonic male normalization^29^ ^-^ ^31^, can alter the patterns of the search for help, response to the treatment and even management of the individual symptoms, in addition to strengthening limiting beliefs that young people are unwavering, resistant and unattainable regarding the psychosocial impacts^28^.

A broader perspective of the mental health concept was observed in the aged men’s CSD, which is related to their experience in the world and is not restrictive to the ailments experienced in the pandemic. Such characteristic can confer greater skills to analyze and face inabilities to deal with new situations and major uncertainties^32^ ^-^ ^33^. Regarding this aspect, it is already known in the literature that aged men are more vulnerable to COVID-19, with very high morbidity and mortality rates^34^. However, in relation to mental health, our results revealed that this population segment is more adherent to and concerned with the disease prevention and control measures, which are closely related to the link with the networks (support, socio-affective, health care), when compared to young men.

Men’s mobilization in the search for self-help interventions in mental health was recognized. This mobilization elucidated the development of “first-aid skills in mental health”^28^, directing them to face the experience in the pandemic, based on the acquisition and construction of knowledge and beliefs. Among the young men, our results verified the use of positive attitudes with a focus on leisure and entertainment and body practices and, among the aged men, maintenance of a balance in mental health, expressed in meditative and self-knowledge practices.

Self-help skills are essential for self-management in mental health, as well as the recognition of when and how to implement them^8^ ^-^ ^11^ ^,^ ^28^. Among the most popular self-help interventions are the search for support from family and friends, engagement in pleasurable activities, initiation of new activities and practice of physical exercise. However, there is limited evidence about the effectiveness of self-help interventions when compared to professional interventions, which makes it difficult to identify which ones are likely to work. However, a study indicates positive results related to physical activity, virtual games and strategies focused on social support and muscle relaxation techniques^35^.

In addition to the self-help skills, it is important that men know the proper time to seek professional help. In this sense, the men revealed a decision-making process based on the search for support and/or professional assistance and of other natures with the intention of providing psychosocial support, offering a space for listening, receiving demands and needs for psychiatric intervention and medicalization, motivated by access to the knowledge that they had and by the beliefs built on the pandemic context and the relationship with mental health. Such attitudes manifested by the men facilitated recognition and search for help to reduce the deleterious impacts caused by the COVID-19 pandemic on mental health, conferring good MHL levels.

Knowledge on how to help others is a component related to MHL^9^ ^-^ ^11^ ^,^ ^28^. A Swiss survey found that the population has difficulty dealing with mental disorders, stating that they do not know how to behave, are afraid of making mistakes, do not have enough knowledge to offer support or have stigmas related to mental health problems^36^. It is understood that public beliefs about professional help can affect the search for help by other people. Professional help for mental health problems is more likely to occur when another person recommends that assistance be sought, so that the opinions of other important people about the treatment can also exert an influence^28^.

In relation to the beliefs of the population and professionals about mental disorders, it is known that the latter have specialized knowledge, largely based on diverse scientific evidence and expert consensus, while the population has a variety of beliefs based on personal experiences, media reports and informal knowledge sources. However, the analysis of the public beliefs does not reveal any general factor corresponding to MHL but a series of factors that, through those beliefs, point out that mental disorders are better treated by medical and psychological interventions or changes in lifestyle^28^.

In relation to the way in which men had knowledge and beliefs about the professional help available for access, it is relevant to highlight the fact that the digital age, of Big Data and algorithmic connections, imprints major transformations in the way in which young and aged men acquire and exercise MHL. In our study, it is evident, for example, that knowledge in computer science is more aggregating and competent among the young men when compared to the aged subjects, making it easier to use tools to maintain affects (meetings, lives and virtual parties, such as the sending directs, erotic messages and access to relationship apps, modeled by geolocation) in the face of physical distancing and social isolation.

In this sense, accessing health information becomes easy for a relatively young sample with potentially high affinity for the online environment, where information is easily accessible at any moment. However, it requires the individual’s ability to filter the diverse information in terms of veracity and precision^37^, with the need to develop higher digital literacy levels^38^ and MHL among the younger populations^39^.

However, it is necessary to consider the presence of complicating factors within MHL, especially among younger men, who may establish a more urgent self-help relationship, in a search for a momentary cure, in order to maintain the pleasures of life, and consequently exposing them to riskier situations for COVID-19 and mental health maintenance: attending clandestine parties, abusive consumption of alcohol and other drugs, pornography, violent and gambling games, experiencing job loss and financial difficulties, and breakups of family and affective bonds^40^ ^-^ ^41^. In turn, among the aged men attention should be paid to strengthening mental health protection strategies and coping with more global psychological disorders (ways of life, family, work, social and affective network), towards adaptation of this target population, in order to build a more consolidated and, possibly, more lasting MHL. Therefore, it presents advances in the understanding that aged men anchor their experiences and practices in the logic that it is fundamental to devote attention to mental health, managing and protecting it. However, it is a scenario that has not yet been explored in depth among men^42^.

The low literacy level in the young and literate population indicates certain vulnerability of people with mental health problems with regard to the perception of difficulties taking care of their own health^37^ ^,^ ^43^. In pandemic times, difficulties can be oversized and establish barriers to understanding, support and coping in the mental health scope. Such MHL levels can be present and cause male invisibility, not placing the mental health position in the transversality of life, which was already historically evidenced among the men’s narratives, due to the oppression systems imposed by *machismo*, by patriarchy and by the social construction of a hegemonic masculinity model^4^ ^,^ ^8^.

It is important to highlight that previous studies have evidenced that low MHL is associated with male gender, advanced age (> 60 years old) and schooling level, suggesting that older adults present a lower mental health literacy level than younger adults, including less precision in identifying symptoms of mental disorders and treatment modalities^44^ ^-^ ^46^. However, our study does not support such conclusions, as it evidenced that aged men, regardless of their schooling level, showed higher MHL levels when compared to younger men. This fact can be explained by the environmental influences, such as proximity to someone who has a mental disorder, which facilitates obtaining adequate information regarding the strategies for promotion, treatment and education in mental health^46^.

When working with subgroups of men, it was evident that structural inequalities in health require political attention from the government to effectively tailor and direct efforts to promote men’s MHL and health promotion programs, particularly with regard to mental health and to adapt the existing health education tools to focus on specific men’s health problems, including depression, suicide and stigma^47^ ^-^ ^49^. The findings of our study only reinforce that knowledge alone does not inform or strengthen men’s MHL in their practices, but indicates the need to make actions more accessible to the male population.

It is highlighted that the CSD herein analyzed evidences that the male population, regardless of their schooling level or age group, has MHL levels that need to be strengthened, considering the important consequences that this insufficient knowledge may reflect. Given this scenario, there is an urgent need to establish joint actions, intersectoral technical cooperation for the massification of the mental health concept, from the expansion of MHL among men in the settings in which they are, as a way of making this population more sensitive and inclined to recognizing themselves as vulnerable and to be able to adopt empowered care self-management measures and free from stigmatizing judgments and beliefs. 

In this sense, as the largest health workforce and part of the Family Health teams in Brazil, Nursing assumes a fundamental role in promoting higher MHL levels in the communities, through dialog and accessible language contextualized with each individual’s reality^50^ ^-^ ^52^.

It is noted that the task of strengthening the MHL levels among men should not only be restricted to the professionals, given the unequal coverage of community services and the scarcity of human resources for mental health care in all regions, as well as the government’s attempt to modify the national mental health policy in a retrograde manner^53^ ^-^ ^54^. For attaining greater gains in prevention, early intervention, self-help, stigma reduction and support from others in the community, it is necessary that the society at large receive adequate training and information on mental health, in which basic knowledge and skills are widely distributed, thus strengthening the MHL levels of the population.

The limitations of this study are concentrated in the operationalization of the technique for recruitment and selection of participants, as well as the possibility of losing inclusion criteria, given the distinct characteristic of the sample (young and aged men), which may have implied the specific race/skin color and social class clipping, in addition to the fact that the sample became concentrated in a given territory and particularity, which had repercussions on the greater apprehension of participants from some Brazilian regions (Northeast and Southeast) and on the difficulty deepening on the data, given the barriers imposed by the web survey. In addition to that, as the survey was online and the form was self-completed, illiterate men did not participate, thus influencing the MHL levels.

## Implications for the practice and research

We emphasize that, to the present day, this is the first study to address MHL in the Brazilian scenario, as well as the only one in the world literature to relate this topic to the COVID-19 pandemic in the male population. The implications for the practice that emerged from this study are related to the dialog of a global Mental Health Action Plan to implement mental health promotion and prevention strategies, as well as to achieve sustainable development goals to reduce illiteracy, promoting gender equality in social relations, reducing impoverishment^55^ ^-^ ^56^, as well as supporting the adoption of strategies that contribute to the strengthening of more resilient societies in the post-pandemic period. Thus, it corroborates with the need to expand the actions directed to men’s health and to the field of study in masculinities and MHL, so essential for advancing the public policies^57^.

It is recommended to conduct future research studies that contribute better understanding, as well as to develop interventions that aim at promoting and expanding each of the MHL components in the male population. To this end, new studies must be carried out to obtain the best possible evidence, using validated assessment instruments and including follow-up periods.

## Conclusion

There is an important distinction in the MHL levels between young and aged men, which made the ability to recognize specific disorders or different types of psychological distress more present among aged men than among young subjects, a situation that is also repeated in relation to the knowledge and beliefs about risk factors and causes, as well as to the knowledge and beliefs about self-help interventions to be adopted within the scope of mental health self-management and protection due to the pandemic context experienced.

The generational category proved to be influential in MHL, when evidencing that aged men expressed knowledge and beliefs on the professional help available for access in the pandemic context, elucidated attitudes related to the practicalities for recognizing mental health problems and also for seeking diverse information related to mental health, when compared to young men, which reveals that, due to insufficient MHL, young men can be more vulnerable to mental illness in the pandemic than aged subjects. It is inferred that aged men may have higher MHL levels due to their history and to the different experiences underwent throughout life that add wisdom and, consequently, expand the MHL levels.
